# Saudi Women’s Knowledge and Attitude toward Cervical Cancer Screening, Treatment, and Prevention: A Cross-Sectional Study in Qassim Region (2018-2019)

**DOI:** 10.31557/APJCP.2019.20.10.2965

**Published:** 2019

**Authors:** Rawan A Alnafisah, Remah A Alsuhaibani, Munirah A Alharbi, Azizah A Alsohaibani, Amal Ahmed Ismai

**Affiliations:** *College of Pharmacy, Qassim University, Unaizah, Saudi Arabia. *

**Keywords:** Cervical cancer, pap smear, knowledge, attitude, Qassim, KSA

## Abstract

**Background:**

Cervical cancer is a preventable and curable disease if early diagnosed; however, most of the cases present late; hence, there is a need to raise the awareness about cervical cancer and to establish screening programs. We aimed to assess the knowledge and attitudes toward screening and to determine the current status of awareness among women.

**Methods:**

We conducted a cross-sectional study among 2,220 Saudi women in the Qassim region using a validated questionnaire that gathers data on socio-demographics, knowledge and attitude domains.

**Results:**

Among the participants, 952 (42.9%) were between 31 and 45 years old; most were married and highly educated 1,754 (79%), and showed moderate knowledge about cervical cancer symptoms, prevention, and screening. The most reported symptom was non-cyclic bleeding by 511 (23%), while prolonged use of contraceptives 289 (13%) was the more selected risk factor by participants. We found that 1,881 (84.7%) of women had not undergone a Pap smear test, and 805 participants (42.8%) expressed their refusal to attend for it, citing various reasons; the most reported was that they do not know someone who tried pap smear. A significant negative correlation was found between the level of knowledge and acceptance to vaccinate daughters at school age against human papillomavirus.

**Conclusion:**

Saudi women in the Qassim region have moderate awareness of cervical cancer but negative attitudes toward screening. Awareness campaigns are needed to promote knowledge and improve pap smear attendance to eliminate negative perceptions and beliefs.

## Introduction

Cervical cancer is a significant global public health problem. The World Health Organization (WHO) reported that cervical cancer is ranked as the fourth most frequent cancer in women (World Health Organization, 2018a). Most cervical premalignant cells develop slowly, so cancer can usually be prevented if a woman is routinely screened (Saslow et al., 2012). Pap smear is a tool to detect these precancerous changes in the uterine cervix. (Al Khudairi et al., 2017).

In a study conducted at Riyadh (Kingdom of Saudi Arabia), 462 (46.2%) of women did not know about cervical cancer screening. While in a study in Bahrain, 320 (64%) of women have known about Pap smear (Jassim et al., 2018; Al Khudairi et al., 2017).

A similar study in Russia found that 80.0% of women had a good level of knowledge about HPV, cervical cancer prevention and screening (Roik et al., 2017).

Cervical cancer is considered a preventable and curable malignancy, but most women in KSA present with advanced stages that need broad treatment modalities, such as chemotherapy, radiotherapy, and surgery, hence the probability of complete remission is significantly diminished (Manji, 2000). Statistics showed that human Papillomavirus (HPV) accounts for 1386 (99%) of all cases (Al-Shaikh et al., 2014).

HPV Information Centre estimates that about 84 Saudi women out of 241 that diagnosed with cervical cancer are dying every year (Sait et al., 2018). There are many risk factors for developing cervical cancer, such as early age onset of sexual intercourse and smoking (Parkin et al., 2001). The United States Preventive Services Task Force (USPSTF) recommends screening for cervical cancer in women (> 21 years old) with pap smear every three years until 29 years old, and every five years women between the ages of 30 and 65 years (pap smear and HPV co-test) (Curry et al., 2018). However, among Muslims, the prevalence of cervical cancer and abnormal smears was lower than in the Western population (Jamal and Al Maghrabi, 2003). Human papillomavirus (HPV) is a sexually transmitted infection and is considered the most important cause of cervical cancer (Parkin et al., 2001). Most sexually active women and men will be infected at some point in their lives, and some may be repeatedly infected. Cervical cancer is considered the most common HPV-related cancer. Almost all cases of cervical cancer can be attributable to HPV infection (World Health Organization, 2018b). The current awareness and attitude about cervical cancer among Saudi women in Qassim has not been previously studied. Also, multiple studies conducted in other regions of the Kingdom of Saudi Arabia (KSA) have revealed poor knowledge of females regarding cervical cancer and screening testing (Malibari, 2018; Sait et al., 2018). The objectives of this study were first to assess the knowledge of Saudi women in the Qassim region about cervical cancer risk factors, symptoms, and pap smear followed by determining their source of knowledge. Moreover, we aimed to evaluate and discuss participant attitude toward screening of cervical cancer.

## Materials and Methods

*Methodology*

A descriptive cross-sectional study was conducted among Saudi women between 15 and 65 years old, residing in the Qassim region (central Saudi Arabia), and agreeing to participate. 

The online self-administrated questionnaire was adapted from the literature and was distributed over two months through social media after being forward-translated into Arabic, back-translated to English, and then double checked by a specialist. It was tested by a pilot study of 40 randomly selected participants who were not involved later in the study.

Questionnaire questions were designed to assess three domains (socio-demographic state, knowledge of cervical cancer screening/treatment/prevention, and attitude towards screening). Internal consistency was measured by assessing the Cronbach’s alpha (0.7). We used a non-probability convenient sampling method in data collection. The sample size was 2,220. Ethics approval was obtained from the research unit committee in Unaizah College of Pharmacy at Qassim University were participants provided written consent that data only will be used for research purposes. 

## Results

Among the 2,220 participants, 952 (42.9%) were between 31 and 45 years old. More than half of the participants were married 1411(63.6%). Two-thirds of responders had completed some higher education. Out of total 1669 (75.2%) denied using a contraceptive, and 111 (5%) reported having a family history of cervical cancer (Table 1).

The majority 1,554 (70%) of participants had previous knowledge about cervical cancer. Only 189 (8.5%) reported that they know of a case of cervical cancer. In addition, 2,122 (95.6%) women agreed that cervical cancer could be treated. Out of all females 2,060 (92.8%) Did not come to know anything about HPV, while half of the participants had heard about the pap smear test. Approximately a third of the women had chosen the right frequency of attending a pap smear. Also, we found 1,523 (68.6%) believes that the HPV vaccine can be useful in the prevention of cervical cancer. Almost all of participants 2,209 (99.5%) did not select ‘direct contact with cancer patients’ as a risk factor. The most selected risk factor was prolonged contraceptive 286 (12.9%) (Table 3).

Non-cyclic bleeding was the most selected symptom 511(23%). Heavy menses, dyspareunia, post-coital bleeding, and excessive vaginal discharge were equally selected by 444 (20%). 

In concern to treatment options 910 (41%) of participants selected ‘combination of surgery, chemotherapy, and radiation’ as the modalities of therapy for cervical cancer.

**Table 1:** Socio-Demographic State (N=2,220)

Variables	Frequency (%)
Age	
15 - 30	921 (41.5)
31 – 45	952 (42.9)
46 – 65	347 (15.6)
Marital status	
Married	1,411 (63.6)
Single	726 (32.7)
Divorced	48 (2.2)
Widow	35 (1.6)
Level of education	
Non-Educated	4 (0.2)
School	503 (22.7)
College	1,670 (75.2)
Postgraduate	43 (1.9)
Are you using a Contraceptive	
Yes	589 (26.5)
No	1,631 (73.5)
Do you have a Family history of cervical cancer	
Yes	110 (5.0)
No	1,640 (73.9)
I do not know	470 (21.2)

**Table 2:** Knowledge about Cervical Cancer (N=2,220)

Variables	Frequency (%)
Did you hear about cervical cancer	
Yes	1553 (70.0)
No	667 (30.0)
Do you know anyone with cervical cancer	
Yes	188 (8.5)
No	2032 (91.5)
Do you think cervical cancer can be treated	
Yes	2122 (95.6)
No	98 (4.4)
Did you hear about human papillomavirus	
Yes	159 (7.2)
No	2061 (92.8)
Did you hear about the pap smear test	
Yes	1165 (52.5)
No	1055 (47.5)
How frequent the test is	
Every year	1259 (56.7)
Every three years	795 (35.8)
Once in a lifetime	166 (7.5)
Do you think the HPV vaccine is useful in the prevention of cervical cancer	
Yes	1524 (68.6)
No	696 (31.4)

**Table 3 A:** Knowledge about Risk Factors for Cervical Cancer (N=2220):

Variables	Frequency (%)
Multiple sex partners	92 (4.1)
HPV	70 (3.2)
HIV	20 (0.9)
Smoking	23 (1.0)
Prolonged usage of contraceptives	286 (12.9)
STDs	60 (2.7)
A positive family history of the disease	149 (6.7)
Early marriage	8 (0.4)
Direct contact with cancer patients	11 (0.5)

**Table 3 B:** Attitudes towards Cervical Cancer Prevention (N=2220)

Variables	Frequency (%)
Did you ever have a pap smear test	
Yes	339 (15.3)
No	1,881 (84.7)
If you did not go for pap previously would you agree to do it Out of 1881	
Yes	1,075 (57.1)
No	805 (42.8)
Do you think screening for cervical cancer is necessary	
Yes	1,825 (82.2)
No	395 (17.8)
Do you agree to provide your daughter the HPV vaccine at school age	
Yes	1,572 (70.8)
No	648 (29.2)

**Figure 1:**
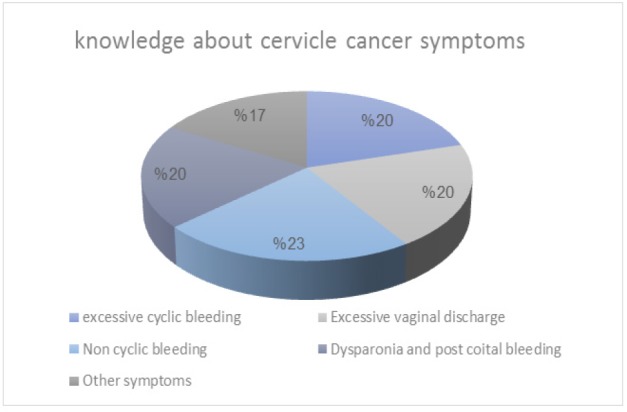
Respondent’s Selection of Cervical Cancer Symptoms

**Figure 2:**
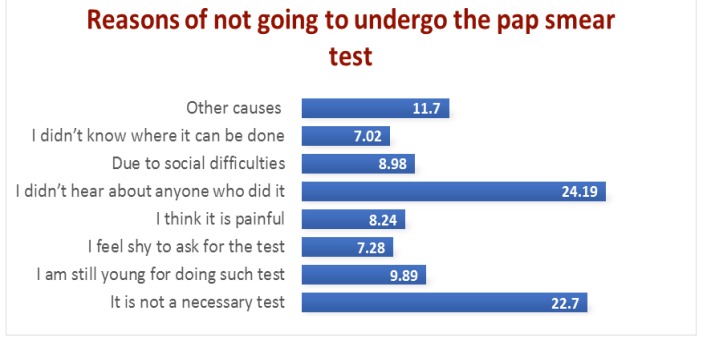
Reasons for Refusing Pap Smear Attendance

Among the participants, 817 (36.8%) reported internet searching as their primary source of knowledge. We found that 1,754 (79%) showed moderate knowledge of cervical cancer and only 89 (4%) good knowledge.

Regarding attitudes, most of the women had never gone for pap smear screening 1,880 (84.7%). Among those participants who had never had a pap smear test, only 1,075 (57.2%) indicated a willingness to undergo it in the future. A significant number of participants 1,825 (82.2%) thought that screening of cervical cancer is necessary. Moreover, 1,572 (70.8%) agreed to provide their daughter with the HPV vaccine at school age (Table 4).

The majority of respondents 537 (24.19%) reported that they had not heard about anyone who had done the pap smear and they believe it is not a necessary test.

On the other hand, the least chosen reason for not having a pap smear was that they did not know where it can be done 156 (7.02%) (Figure 2).

## Discussion

Cervical cancer is preventable malignancy but requires knowledge about the disease and prevention parameters. Our study was the first study addressing cervical cancer conducted in the Qassim region. Our population sample was large in comparison to other studies conducted in Saudi Arabia. (Malibari, 2018; Al Shaman et al., 2016; Al-Shaikh et al., 2017; Al Khudairi et al., 2017; Jassim et al., 2018; Almansour et al., 2018).

The results of our cross-sectional study reported a moderate level of knowledge about cervical cancer, presenting symptoms, predisposing risk factors, treatment options, the possibility of prevention, and pap smear. In our study, we found more than 1,554 (70%) had previous knowledge of cervical cancer which is higher than previous studies done in Saudi Arabia; 170 (67.9%) at Tabuk University in KSA. (Al Shaman et al., 2016), and 217 (43.3%) reported by Almansour et al., (2018) in hail. Scoring of knowledge showed a moderate level of knowledge similar to the study conducted in Uganda (Mukama et al., 2017). The high educational level of our participants 694 (77.1%) might account for the high proportion that was knowledgeable. The majority (73.5%) reported that they are not using contraceptives. The prolonged use of contraceptives was the more selected risk factor for cervical cancer by the participants; this result reflects a piece of knowledge about risk factors and explains the negative attitude toward contraceptives administration. Although HPV is considered the most important risk factor, we found that 2,260 (92.8%) did not hear about it, and HPV was selected as a risk factor only by 71 (3.2%), this result was similar to findings of Al-Shaikh et al., (2014). 

Regarding symptoms of cervical cancer, the most selected choice was noncyclic bleeding, which is in line with the findings of the Tabuk university study, while abdominal pain was the most selected symptom in the Mukama et al., (2017) study.

We noticed that 817 (36.8%) stated that their knowledge was based on internet searches. Health care providers were the source of only 100 (4.5%) of the participants’ knowledge this finding was relatively similar to Al Shaman et al., (2016) study in which most of the respondents reported the media as their source of knowledge in comparison to the study in Bahrain that reported gynecologists 145 (51.5%) (Jassim et al., 2018) as the primary source of information. It is clear that health authorities need to improve awareness of the role of health care providers in cervical cancer screening. 

Only 340 (15.3%) of the respondents had a pap smear test; this is far less than the percentages reported in previous studies in Saudi Arabia at Umm Al-Qura University, United Arab of Emirates, Bahrain, and Qatar. (Al Shaman et al., 2016; Mukama at al., 2017; Al-Meer et al., 2011). However, our finding is similar to the percentages reported in similar studies in Hail (Malibari, 2018).

Reasons for not attending a pap smear differed among respondents, with approximately one-quarter (24.19%) reporting that they had not heard about anyone who had done the test. Bahraini women in the study conducted at primary health centers stated that the Pap smear procedure was unpleasant or embarrassing (Jassim et al., 2018)

Most 1,572 (70.8%) of the total participating women answered yes to the question ‘do you agree to provide the HPV vaccine to your daughter at school age,’ but this is lower than the proportion reported by Bahrain 455 (90.9%). The negative statistical correlation between the participant’s level of knowledge and their agreement of vaccinating daughters reveals that the decision is not stated from their knowledge about vaccination importance but may be due to their positive attitude toward vaccination.

In conclusion, we conclude that Saudi women in the Qassim region have moderate awareness of cervical cancer but negative attitudes toward screening. These attitudes reflect traditions, emotions, and culture of the region. These findings will help health workers in this field when designing, targeting, and implementing public health programs for cervical cancer prevention. We recommend that awareness campaigns are used to promote knowledge and eliminate negative perceptions and beliefs, thereby improving pap smear attendance. Screening services should be incorporated into the existing health care system. Moreover, to obtain more generalizable results, we suggest replication of the study on a larger sample selected from different multiple areas in KSA.
